# Evidence of recent interspecies horizontal gene transfer regarding nucleopolyhedrovirus infection of *Spodoptera frugiperda*

**DOI:** 10.1186/s12864-015-2218-5

**Published:** 2015-11-25

**Authors:** Gloria Patricia Barrera, Mariano Nicolás Belaich, Manuel Alfonso Patarroyo, Laura Fernanda Villamizar, Pablo Daniel Ghiringhelli

**Affiliations:** Centro de Investigación Tibaitatá, Corpoica (Corporación Colombiana de Investigación Agropecuaria), Km 14 Vía Mosquera, Cundinamarca, Colombia; Laboratorio de Ingeniería Genética y Biología Celular y Molecular – Área Virosis de Insectos (LIGBCM-AVI), Dto. Ciencia y Tecnología, Universidad Nacional de Quilmes, Roque Sáenz Peña 352, B1876BXD Bernal, Buenos Aires Argentina; Departamento de Biología Molecular e Inmunología, Fundación Instituto de Inmunología de Colombia (FIDIC), Avenida 50 N° 26-20, Bogotá, Colombia; Departamento de Ciencias Básicas, Escuela de Medicina y Ciencias de la Salud, Universidad del Rosario, Calle 12C N° 6-25, Bogotá, Colombia

**Keywords:** Baculovirus, *Spodoptera frugiperda*, Horizontal gene transfer, Homologous recombination

## Abstract

**Background:**

Baculoviruses are insect-associated viruses carrying large, circular double-stranded-DNA genomes with significant biotechnological applications such as biological pest control, recombinant protein production, gene delivery in mammals and as a model of DNA genome evolution. These pathogens infect insects from the orders Lepidoptera, Hymenoptera and Diptera, and have high species diversity which is expressed in their diverse biological properties including morphology, virulence or pathogenicity. *Spodoptera frugiperda* (Lepidoptera: Noctuidae), the fall armyworm, represents a significant pest for agriculture in America; it is a host for baculoviruses such as the *Spodoptera frugiperda* multiple nucleopolyhedrovirus (SfMNPV) (Colombia strain, genotype A) having been classified as a Group II alphabaculovirus making it a very attractive target for bioinsecticidal use.

**Results:**

Genome analysis by pyrosequencing revealed that SfMNPV ColA has 145 ORFs, 2 of which were not present in the other sequenced genotypes of the virus (SfMNPV-NicB, SfMNPV-NicG, SfMNPV-19 and SfMNPV-3AP2). An in-depth bioinformatics study showed that ORF023 and ORF024 were acquired by a recent homologous recombination process between *Spodoptera frugiperda* and *Spodoptera litura* (the Oriental leafworm moth) nucleopolyhedroviruses*.* Auxiliary genes are numerous in the affected *locus* which has a homologous region (*hr*3), a repetitive sequence associated with genome replication which became lost in SfColA along with 1 ORF. Besides, the mRNAs associated with two acquired genes appeared in the virus’ life-cycle during the larval stage. Predictive studies concerning the theoretical proteins identified that ORF023 protein would be a phosphatase involved in DNA repair and that the ORF024 protein would be a membrane polypeptide associated with cell transport.

**Conclusions:**

The SfColA genome was thus revealed to be a natural recombinant virus showing evidence of recent horizontal gene transfer between different baculovirus species occurring in nature. This feature could be the cause of its high insecticidal power and therefore SfColA becomes a great candidate for bioinsecticide formulations.

**Electronic supplementary material:**

The online version of this article (doi:10.1186/s12864-015-2218-5) contains supplementary material, which is available to authorized users.

## Background

Baculoviruses are double-stranded DNA viruses which infect insects from the orders Lepidoptera, Diptera and Hymenoptera. Significant baculovirus characteristics include the presence of two phenotypes during the cell cycle: budded viruses (BVs), and occlusion derived viruses (ODVs) which are embedded into protein crystals called occlusion bodies (OBs); they display very high diversity expressed in hundreds of species spread worldwide having different host ranges and/or virulence. Four genera have been recognized: *Alphabaculovirus* (lepidopteran-nucleopolyhedroviruses), *Betabaculovirus* (lepidopteran-granuloviruses), *Gammabaculovirus* (hymenopteran-nucleopolyhedroviruses), and *Deltabaculovirus* (dipteran-nucleopolyhedroviruses) [1–3]. The *Spodoptera frugiperda* multiple nucleopolyhedrovirus (SfMNPV) has been classified into the *Baculoviridae* family within the *Alphabaculovirus* [1, 4] and has been extensively studied for its potential regarding the biological control of fall armyworm, an important pest causing economic losses regarding several American crops, mainly corn fields [5]. Since the first reports about SfMNPV [6, 7], work has been focused on studying its genomic constitution and the inter- and intra-population diversity by comparing different isolates. Restriction profiles initially revealed genetic heterogeneity in field isolates, in addition to providing information for determining the first physical maps of the genome [8–10]. Sequencing of single genes and genomic variable regions [11–15] and subsequent analysis showed that SfMNPV phylogenetically clustered with other members of the Group II *Alphabaculovirus* clade [1].

Four complete SfMNPV genomes have been reported recently, one from a field isolate (SfMNPV-19) [16] and another three from genotypic variants recovered using *in vitro* plaque assay techniques in insects cells (SfMNPV-3AP2, SfMNPV-NicB, SfMNPV-NicG) [17–19]. The SfMNPV-NicB isolate was the predominant genotype having the largest genome and both SfMNPV-3AP2 and SfMNPV-NicG had deletions regarding the former. Phylogenetic analysis revealed that they were very closely related and also closely related to *Spodoptera exigua* MNPV, *Agrotis spp*. NPVs and *Mamestra spp*. NPVs.

SfMNPV inter-population diversity evaluated in Colombia by analyzing 38 isolates from three different geographical regions revealed that one isolate (SfMNPV-Col or SfCol) had the highest prevalence (92 %). SfCol had minimal genetic differences compared to the SfMNPV isolate from Nicaragua (SfMNPV-NicB or SfNicB) based on restriction profiles; however, it had large differences regarding virulence against *S. frugiperda* larvae from Colombia, SfCol being more potent than SfNicB for the local insect population [20]. Subsequent intra-population diversity studies have revealed 10 different genotypic variants within SfCol (SfColA to SfColJ), SfColA being the most prevalent (72 %) and having the largest genome, while the other variants had different sized deletions. SfColA was 4.4 times more potent than and as virulent as SfCol for local insect pests [21]. Such biological differences should correlate with genome organization; structural mutations (replacements, inversions, insertions or deletions) would presumably be how baculoviruses evolve in nature and improve their fitness, not forgetting the importance of single nucleotide mutations. According to previous genome evidence, natural recombination events are probably one of the most important processes involved in baculovirus genome plasticity [22]. DNA crossover may occur between two *loci* from one genome, between genotype variants of the same species, or between genomes from different virus species co-infecting the same host [23]. In any case, the resulting recombinant genomes may be affected by their prior gene content. Baculovirus diversity has been associated with the ubiquitous presence of transposons, which may collaborate in horizontal gene transfer and insertion/deletion (indel) mutations. Different kinds of transposable elements have been detected in baculovirus genomes from many species, sometimes affecting gene functions [24, 25]. Baculoviruses should thus be efficient vectors between animals and such ability would have an important impact on gene content and genome organization because they can provide the sequence homology required for crossover events [26].

Baculovirus genome variability has an undeniable effect on the virus’ life-cycle in the host and affects different parameters such as pathogenicity, virulence and OB’s production (yield) [27, 28]. Some genome regions are more prone to sequence variation than others; these would include *loci* containing homologous regions (*hr)* and *Bro* genes, both being the kind of sequences recognized as target sites for intragenomic recombination because they are usually found in more than one copy [28]. Moreover, most variability is concentrated in regions having auxiliary genes (encoding non-essential proteins) as they are more tolerant to mutations because sequence changes do not affect the production of essential factors needed to complete the viral cycle.

The SfColA isolate was molecularly characterized in the present work to provide extra evidence to explain biological activities and to further understand how baculoviruses evolve in nature, losing ancient sequences or gaining new regions and thereby altering virus fitness.

## Methods

### Virus isolate

The virus used here [SfMNPV ColA (SfColA)] had been previously isolated by plaque purification in the Sf9 cell line exposed to a natural SfMNPV isolated in Colombia (SfMNPV-Col or SFCol) [21]. SfColA was propagated in *S. frugiperda* fourth instar larvae reared in laboratory conditions (25 ± 1 °C, 75 ± 5 % relative humidity, 16 h light: 8 h dark photoperiod and a wheat germ-based semisynthetic diet) and maintained as OB suspension in sterile distilled water.

### Sequencing, assembly and ORFeome determination

SfColA DNA was purified from OBs by alkaline lysis and cesium chloride gradient [29] and used for sequencing with the 454 Genome Sequencer (GS) FLX™ Standard (Roche) (Centro Nacional de Secuenciación Genómica, CNSG; Universidad de Antioquia, Medellín, Colombia). *De novo* assembly was performed using NewBler assembler (GS FLX Data Analysis Software) to define whole genome sequence. The reads were independently assembled five times without using a reference genome; in all the runs the resulting sequence was essentially the same. This assembly was then compared to the genomes from baculoviruses which infect *Spodoptera* spp. (SfMNPV-3AP2, SfMNPV-NicB, SfMNPV-NicG, SeMNPV and SpltNPV-II) and the genome organisation was conserved, thereby validating the previous result. The SfMNPV-ColA assembly correlated with experimental physical map data and the region containing differential genes was confirmed by Sanger sequencing. Open reading frames (ORFs) were identified using ARTEMIS [30]. ATG initiated ORFs having at least 150 nt (50 aa) showing minimal overlap with other putative encoding sequences were selected for further analysis. BlastN, BlastP, tBlastN, tBlastX and PSI-Blast were used for homology searches [31], initially against other SfMNPV genomes and then against other baculovirus species. Homologous genes’ identity and similarity values were obtained by global alignment using ClustalX [32, 33] with default parameters. The baculovirus genomic sequences used in the bioinformatics studies are listed in Table 1.

**Table 1:** Baculoviruses used in bioinformatics studies including SfMNPV ColA

*Baculovirus*	Acc. number	Abbreviation
*Antheraea pernyi* MNPV Isolate L2	EF207986	AnpeMNPV
*Antheraea pernyi* NPV Isolate Z	NC_008035	AnpeNPV
*Anticarsia gemmatalis* MNPV	NC_008520	AgMNPV
*Autographa californica* MNPV Clone C6	NC_001623	AcMNPV
*Bombyx mandarina* NPV S1	NC_012672	BomaNPV S1
*Bombyx mandarina* NPV S2	JQ071499	BomaNPV S2
*Bombyx mori* NPV Isolate T3	NC_001962	BmNPV
*Choristoneura fumiferana* MNPV	NC_004778	CfMNPV
*Choristoneura fumiferana* Defective MNPV	NC_005137	CfDEFMNPV
*Choristoneura murinana* NPV Strain Darmstadt	NC_023177	ChmuNPV
*Choristoneura occidentalis* NPV Isolate BC1	NC_021925	ChocNPV
*Choristoneura rosaceana* NPV Isolate NB1	NC_021924	ChroNPV
*Epiphyas postvittana* NPV	NC_003083	EppoNPV
*Hyphantria cunea* NPV	NC_007767	HycuNPV
*Maruca vitrata* NPV	NC_008725	MaviNPV
*Orgyia pseudotsugata* MNPV	NC_001875	OpMNPV
*Philosamia cynthia ricini* NPV	JX404026	PhcyNPV
*Plutella xylostella* MNPV Isolate CL3	NC_008349	PlxyMNPV
*Rachiplusia ou* MNPV	NC_004323	RoMNPV
*Thysanoplusia orichalcea* NPV P2	NC_019945	ThorNPV P2
*Adoxophyes honmai* NPV	NC_004690	AdhoNPV
*Adoxophyes orana* NPV	NC_011423	AdorNPV
*Agrotis ipsilon* MNPV	NC_011345	AgipMNPV
*Agrotis segetum* NPV	NC_007921	AgseNPV
*Apocheima cinerarium* NPV	NC_018504	ApciNPV
*Buzura suppressaria* NPV Isolate Hubei	NC_023442	BusuNPV
*Chrysodeixis chalcites* NPV	NC_007151	ChchNPV
*Clanis bilineata* NPV Isolate DZ1	NC_008293	ClbiNPV
*Ectropis obliqua* NPV Strain A1	NC_008586	EcobNPV
*Euproctis pseudoconspersa* NPV	NC_012639	EupsNPV
*Helicoverpa armigera* MNPV	NC_011615	HearMNPV
*Helicoverpa armigera* NPV Isolate Australia	JN584482	HearNPV Aus
*Helicoverpa armigera* NPV Strain C1	NC_003094	HearNPV C1
*Helicoverpa armigera* NPV Strain G4	NC_002654	HearNPV G4
*Helicoverpa armigera* SNPV Strain NNg1	NC_011354	HearSNPV
*Helicoverpa zea* NPV	NC_003349	HzSNPV
*Hemileuca* sp. NPV	NC_021923	HespNPV
*Leucania separata* NPV Strain AH1	NC_008348	LeseNPV
*Lymantria dispar* MNPV	NC_001973	LdMNPV
*Lymantria xylina* MNPV	NC_013953	LyxyMNPV
*Mamestra brassicae* MNPV Isolate Chb1	JX138237	MabrMNPV Chb1
*Mamestra brassicae* MNPV Isolate K1	NC_023681	MabrMNPV K1
*Mamestra configurata* NPV Strain 90-2	NC_003529	MacoNPV 90 2
*Mamestra configurata* NPV Strain A90-4	AF539999	MacoNPV A90 4
*Mamestra configurata* NPV Strain B	NC_004117	MacoNPV B
*Orgyia leucostigma* NPV IsolateCFS77	NC_010276	OrleNPV
*Spodoptera exigua* MNPV	NC_002169	SeMNPV
*Spodoptera frugiperda* MNPV Isolate 3AP2	NC_009011	SfMNPV 3AP2
*Spodoptera frugiperda* MNPV Isolate Nicaraguan B	HM595733	SfMNPV NicB
*Spodoptera frugiperda* MNPV Isolate Nicaraguan DefG	JF899325	SfMNPV NicG
*Spodoptera frugiperda* MNPV Strain 19	EU258200	SfMNPV 19
*Spodoptera frugiperda* MNPV ColA	KF891883	SfMNPV ColA
*Spodoptera littoralis* NPV Isolate AN1956	JX454574	SpliMNPV AN1956
*Spodoptera litura* II MNPV	NC_011616	SpltNPV-II
*Spodoptera litura* MNPV Strain G2	NC_003102	SpltMNPV G2
*Trichoplusia ni* SNPV	NC_007383	TnSNPV
*Adoxophyes orana GV*	NC_005038	AdorGV
*Agrotis segetum GV*	NC_005839	AgseGV
*Choristoneura occidentalis GV*	NC_008168	ChocGV
*Clostera anastomosis* GV Strain Henan	NC_022646	CaLGV
*Cryptophlebia leucotreta GV*	NC_005068	CrleGV
*Cydia pomonella GV*	NC_002816	CpGV
*Epinotia aporema* GV	NC_018875	EpapGV
*Helicoverpa armigera GV*	NC_010240	HearGV
*Phthorimaea operculella GV*	NC_004062	PhopGV
*Pseudaletia unipuncta* GV Strain Hawaiin	NC_013772	PsunGV
*Pieris rapae GV*	NC_013797	PiraGV
*Plutella xylostella GV*	NC_002593	PlxyGV
*Spodoptera frugiperda* GV	KM371112	SpfrGV
*Spodoptera litura* GV Strain K1	NC_009503	SpliGV
*Xestia c-nigrum* GV	NC_002331	XecnGV
*Neodiprion abietis* NPV	DQ317692	NeabNPV
*Neodiprion lecontei* NPV	NC_005906	NeleNPV
*Neodiprion sertifer* NPV	NC_005905	NeseNPV
*Culex nigripalpus* NPV	NC_003084	CuniNPV

For detecting *homologous regions* (*hr*s) in the SfColA genome, the SfMNPV NicB *hr-1* sequence was used as computational probe. All individual palindromes (44 residue lengths) were then recovered from SfColA, SfMNPV Nic, SfMNPV 3AP2 and SfMNPV 19 genomes and multiple alignments were performed using the ClustalX algorithm with default parameters. Sequence logos were constructed using the WebLogo server (http://weblogo.berkeley.edu/) [34]. The secondary DNA structure prediction was obtained using the Mfold server of Michael Zuker website [35] and using RNADraw program [36]. A/T-content was profiled using a sliding windows strategy (window = 500 nucleotides, displacement = 50 nucleotides) [37]. Relationships between each point and the A/T-content average were obtained and peaks of 1.12 or above were considered as A/T-rich regions.

### Colinearity genome studies and phylogenetic analysis

Nucleotide synteny blocks were searched using BlastN routine with the following parameters: expected value = 0.1 (−e 0.1), penalty for a nucleotide mismatch = −2 (−q −2), reward for a nucleotide match = 1 (−r 1) and filter query sequence = false (−F F). Output files for each genome comparison were drawn using the GenomeComp v1.2 software [38]. A color code was used for showing different ranges of nucleotide identity. *Baculoviridae* phylogeny was inferred using the 37 core genes *in silico* translated from 75 baculovirus genomes (Table 1) which were independently aligned using ClustalX program with the following parameters: Pairwise alignment (Gap Open Penalty = 10, Gap Extension Penalty = 0.1, protein weight matrix: Blosum 30); Multiple alignment (Gap Open Penalty = 10, Gap Extension Penalty = 0.05, protein weight matrix: Blosum series). A concatemer was then generated by adding complete individual alignments and phylogeny was inferred using MEGA 5 software [39] with the following parameters: UPGMA; Bootstrap with 1000 replicates; Gap/Missing data = pairwise deletion; Model = Amino (Dayhoff Matrix); patterns among sites = Same (Homogeneous); rates among sites = Different (Gamma Distributed); gamma parameter = 0.9839. Besides, a phylogeny inference was similarly performed but using only SfMNPV genomes and the most related baculoviruses (SeMNPV, SpltNPV-II, SpltMNPV-G2, SpliNPV AN1956). The concatemer of individual alignments derived from 100 genes translated *in silico* which were shared among baculoviruses considered for the study (indicated in Additional file 1: Table S1).

### Interspecies horizontal gene transfer studies

The partial SfColA genome sequence, from *chitinase* ATG to the *gp37* stop codon genes, was compared to corresponding SfMNPV-B, SfMNPV 3AP2, SfMNPV 19, SeMNPV and SpltNPV-II regions to detect potential recombination events by running alternative methods. In the first one [37], ClustalX (default parameters) was used for aligning sequence pairs, always involving the putative recombinant candidate (SfColA) and one of the other sequences. Relative similarities were calculated using the ClustalX consensus symbol (* and blank space) as the input sequence, in an overlapping windows-based strategy. Arbitrary values of +1 for identical (*) and −1 for non-identical residues (blank spaces) were set for obtaining similarity profiles. The sum of assigned values for each residue in each window (35 nucleotides) was divided by the window width and allotted to the central position to generate the plots. Profiles were drawn and analyzed for detecting crossover points. Different window lengths were scanned to find good relationships between graph complexity and crosspoint detection sensitivity. Bootscan analysis (Simplot program, version 3.5.1) [40, 41] was performed using the following parameters: Window = 500 residues; Step = 50 residues; Gaps strip = on; Replicates = 100; Model = Kimura 2-parameters; Transition and transversion ratio = 2.0; Phylogenetic method = Neighbor Joining). A G/C-content study was made for the same SfColA, SfNicB and SpltNPV-II genome region. G/C-contents were studied using a sliding window strategy (window = 65 nucleotides, displacement = 1 nucleotide). The profiles were adjusted using in-house software. For this a multiple alignment with the three nucleotide sequences was used as template and the adjustment of numerical profiles consisted in the introduction of blank positions interrupting the curve where there were indel events. SigmaPlot v9 was used for all studies where final numerical profiles were represented and the putative crossover breakpoints were estimated. Multiple alignment of homologous proteins was done to estimate Kimura 2-parameter distances [42] using MEGA 5 software with the following parameters: Scope = Pairs of taxa; Estimate Variance, Variance Estimation Method = Bootstrap method; N° of Bootstrap Replications = 1000; Substitution model, Substitution type = Nucleotide, Model/Method = Kimura 2-parameter model, Substitutions to include = d: Transitions + Transversions; Rates and Patterns, Rates among Sites = Gamma parameter, Gamma Parameter = 0.9839, Pattern among lineages = Same (Homogeneous); Data Subset to Use, Gaps/Missing Data Treatment = Pairwise deletion, Select Codon Positions = All + Noncoding Sites.

### SfColA ORF023 and ORF024 transcription studies

Reverse transcription-PCR assays were performed to determine SfColA ORF023 and ORF024 transcription activities. Forty five *S. frugiperda* second-instar larvae were kept fasting for 8 to 12 h at 26 °C and then allowed to drink from an aqueous suspension containing 10 % (wt/vol) sucrose, 0.001 % (wt/vol) Fluorella blue, and 1x10^7^ OBs/mL. Larvae that ingested the suspension within 10 min were then transferred to individual plastic cups with semisynthetic diet. Total RNA was extracted from two infected larvae at 0, 2, 4, 6, 12, 24, 48, 72, 96, 120 and 144 hpi using TRIzol reagent (Invitrogen), as described in the manufacturers’ protocol. RNA samples were then treated with RNase free DNase (Promega) prior to ensuring the absence of contaminant DNA. First strand cDNA synthesis was done using reverse transcriptase (Promega) and oligo-dT primer. The resulting cDNAs were amplified by PCR with specific primers: Sf23.1 (5′ GCTTGTGCGTTGTCGTTGAT 3′) and Sf23.2 (5′ TTGTAGTCGACTCGGTCCCA 3′) for ORF023; Sf24.1 (5′ TCGTCGGCATCATACTGCTC 3′) and Sf24.2 (5′ CACGTTCGCATGGTTTTCGT 3′) for ORF024; Sfpolh.1 (5^′^ TTGCGACCCTGACTACGTTC 3^′^) and Sfpolh.2 (5^′^ ACGAGCGACAGTTCAAGGAG 3^′^) for the very late transcribed polyhedrin gene (*polh*); Sfie-0.1 (5^′^ CATTTGCCAAGAGAGCAGCG 3^′^) and Sfie-0.2 (5^′^ TTTAGCGGCAGTGGGAGTTT 3^′^) for the early transcribed *ie-o* gene. The amplification products were resolved in 1 % agarose gels and visualized with ethidium bromide and UV exposure.

### Characterization of SfColA ORF023 and ORF024 proteins

Different bioinformatics tools were used for determining the nature of SfColA ORF023 and ORF024 proteins. Hydrophobicity profiles were constructed using a sliding windows strategy (window = 21 amino acids; sliding 1 residue each time). Several hydrophobicity scales were assayed [43–47]. The presence of signal peptides was assessed by using SignalP (http://www.cbs.dtu.dk/services/SignalP/; [48]). Putative functions were predicted using the HHpred server (http://toolkit.lmb.uni-muenchen.de/hhpred; [49]). Secondary and tertiary structures were predicted using the LOcal MEta-Threading-Server (LOMETS; http://zhanglab.ccmb.med.umich.edu/LOMETS; [50]) and the I-TASSER server (http://zhanglab.ccmb.med.umich.edu/I-TASSER; [51]). SfColA ORF023 secondary and tertiary structures were also predicted using the QUARK (http://zhanglab.ccmb.med.umich.edu/QUARK; [52]) *ab initio* prediction server. Post-translational modifications were predicted by the INTERPROSCAN tool (http://www.ebi.ac.uk/interpro/; [53]).

## Results and discussion

### The SfColA genome and gene content

Five genomes have been sequenced to date from baculoviruses isolated from *Spodoptera frugiperda*: 4 alphabaculoviruses (SfMNPV 3AP2, SfMNPV NicB, SfMNPV NicG, SfMNPV 19) and 1 betabaculovirus (SpfrGV) (Table 1)*.* The aforementioned polyhedroviruses were sequenced using a molecular cloning strategy followed by an automated Sanger’s method; only the granulovirus involved using next generation-sequencing (NGS) [37]. The Colombian isolate’s (SfCol) SfColA genotypic variant was molecularly characterized in view of its interesting biological properties to complete the study of the baculovirus group naturally infecting the armyworm and to provide extra information about baculovirus evolution. This genome (GenBank: KF891883) consisted of 134,239 bp, and was covered 64 times by 454 sequencing, this being the first *Spodoptera frugiperda* alphabaculovirus studied by the NGS approach. Its size was revealed to be the largest regarding previously reported genomes where SfMNPV-NicB (132,954 bp) was the previous head of the ranking. Group II alphabaculoviruses have a broad range of A + T content [42.53 % in *Lymantria dispar* MNPV [54] to 66.64 % in *Apocheima cinerarium* NPV (unpublished data)]. SfColA had 59.66 %, thereby agreeing with data published for the other *Spodoptera frugiperda* nucleopolyhedrovirus [SfNicB 59.72 %, SfMNPV-19 (Sf19) 59.74 %, and SfMNPV-3AP2 (Sf3AP2) 59.75 %].

Analysis of the SfColA genome led to detection of 145 putative open reading frames (ORFs) considering sequences encoding polypeptides having at least 50 amino acids, starting in an ATG codon and having minimal overlap with the closest ones. Thus the ORFeome would cover 92.5 % of the whole nucleotide sequence and each ORF was numbered, starting at the polyhedrin gene (ORF001) and continuing the numbering downstream polyhedrin stop codon. Promoter motifs were searched, an early CAKT initiator sequence (INR) [55] was found in 40 ORFs, irrespective of including the TATA-box, while 29 ORFs had a late INR motif [56] and another 59 had both early and late elements. As expected, the SfColA genome contained the 37 core genes present in all baculoviruses and these sequences were thus identified using current denominations. The other putative genes were mentioned using the most accepted names based on their Blast relationships regarding the annotated ORFs from other baculoviruses [3, 57, 58].

**Fig. 1:**
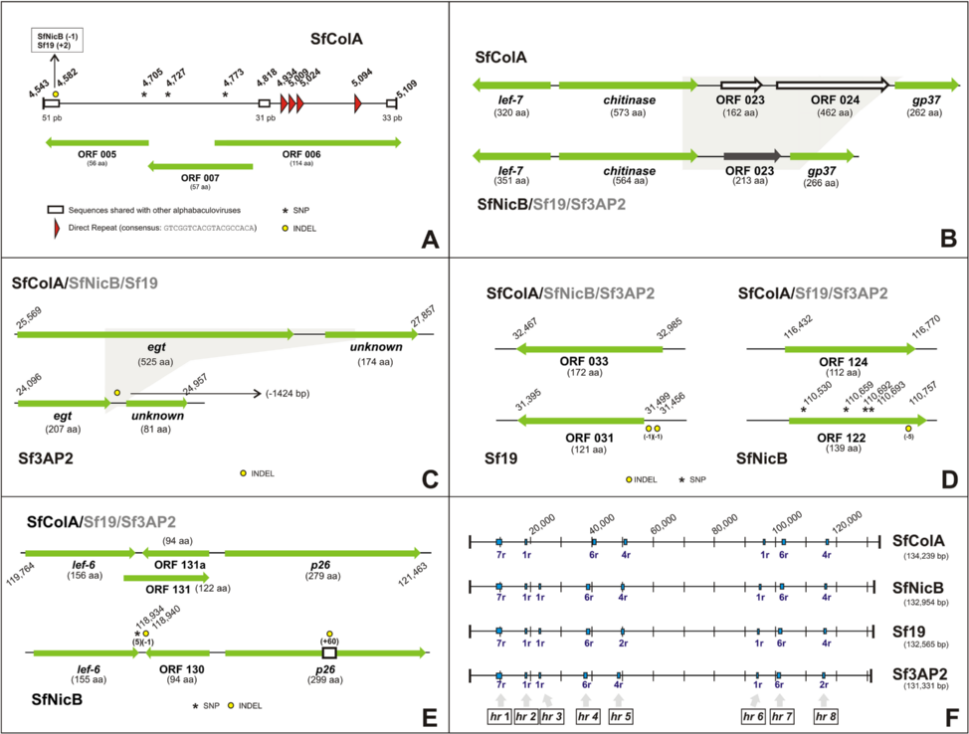
Genome organization of SfMNPV ColA. The illustrations show the SfColA *loci* where there are differences regarding other genotypes of this baculovirus species [SfMNPV NicB (SfNicB), SfMNPV 19 (Sf19) and SfMNPV 3AP2 (Sf3AP2)]. In all cases, the SNPs (single nucleotide polymorphisms; asterisks), indels (sequence insertion-deletions; filled circles indicating in parenthesis the number of nucleotides added or deleted) and the annotated ORFs (shown as arrows) are highlighted in each *locus*. **a** Region containing SfColA ORF005/006/007. White boxes indicate sequences shared by alphabaculoviruses and direct repeats are shown as red triangles. **b** Region containing SfColA ORF023/024. Sequences involved in gene replacement are shaded and the respective ORFs located in that position are differentially colored (white in SfColA and black in the other ones). In Sf3AP2 the ORF023 ortholog of SfNicB and Sf19 is annotated as ORF022. **c** Region containing *egt* gene in SfColA, SfNicB and Sf19. The *unknown* gene annotated in all genomes downstream to the *egt* gene is ORF028 in SfColA, ORF027 in SfNicB, ORF026 in Sf19 and ORF027 in Sf3AP2. The deleted region in Sf3AP2 is shaded in grey. **d** Regions containing SfColA ORF033 and ORF124. The orthologs of the first one in SfNicB and Sf3AP2 are annotated as ORF032. The orthologs of the second sequence in Sf19 and Sf3AP2 are annotated as ORF121 and ORF122, respectively. **e** Region containing SfColA ORF131 and ORF131a. The orthologs in Sf19 and Sf3AP2 are ORF 127a/128 and ORF 129/130, respectively. Four SNPs and a deletion determined the absence of a coding sequence equivalent to SfColA ORF131 in the genome of SfNicB. The *p26* gene in SfNicB has an insertion of 60 nt. **f** Genome representations of homologous region distribution

Sequence homology analysis revealed that most ORFs were shared among SfMNPVs, giving close to 100 % similarity but also revealing significant differences in one set of *loci* (Additional file 1: Table S1). SfColA 005/007/023/024/033/112/124/131 ORFs particularly required more in-depth study because they had less similarity than expected for genotype variants from the same species (having values less than 75 % when the identity average is 98.5 % ± 5.5) or absence regarding their putative orthologs. The region including 005/006/007 ORFs might thus be a putative encoding location for expressing 3 small polypeptides annotated on only SfMNPV genomes (Fig. 1a). It is worth noting that ORF007 is overlapped with ORF006 and probably is not a gene considering the absence of typical promoter motifs and the small size of its theoretical encoded polypeptide. The proteins derived from ORFs 005 and 006 had variability when compared to their orthologs from the other SfMNPVs due to mainly single or double nucleotide insertion-deletions causing frame shifts. What was striking about this unique SfMNPV region was the presence of direct and inverted repeats located on the flanks of a sequence shared with other Group II alphabaculoviruses. This region might thus be considered as non-coding (and until there is experimental evidence) and could be associated with other viral functions such as genomic replication where this kind of sequence seems to be relevant [3]. SfColA ORFs 005/006/007 were annotated since the same was done in SfNicB, Sf19 and Sf3AP2.

A very different situation occurred with SfColA ORFs 023 and 024 (Fig. 1b); both genes were not present in the other SfMNPV genomes, although these sequences have similarity with some Group II alphabaculovirus genes. By contrast, SfNicB, Sf19 and Sf3AP2 had one ORF in that *locus* (023, 022 and 023, respectively) that is not present in SfColA and had homology with other baculoviruses. This gene is present in the SpfrGV genome (ORF099) where the encoded polypeptide was hypothesized as being a soluble protein containing ring finger motifs [37]. Such genome replacement (2 genes acquired compared to 1 lost) might thus be considered a recombination product as will be shown below.

Another genome location having differences was the region containing SfColA ORFs 027 and 028 because the theoretical polypeptides encoded by these genes had low identity and similarity values regarding the homologous Sf3AP2 ORFs 026 and 027 (Fig. 1c). This was due to deletion in the Sf3AP2 genome affecting the corresponding carboxy terminal of the *ecdysteroid UDP–glucosyltransferase* (*egt*) gene (Sf3AP2 ORF026) and the amino terminal of the other one (Sf3AP2 ORF027).

SfColA ORFs 033 and 124 also had lower similarity values than the expected ones when the *in silico* translated sequences were compared to the corresponding orthologs (Fig. 1d). Both putative genes encoded unknown proteins; the former only had differences with Sf19 because of a two single nucleotide deletion in this gene affected the reading frame annotated in the other SfMNPVs starting in a later ATG. SfColA ORF124 had differences with only SfNicB due to this sequence having a 5 bp microdeletion. The SfColA ORF131 did not present an annotated ortholog in SfNicB (Fig. 1e). Sequence analysis revealed 6 different nucleotides in the same stretch, including 1 nucleotide deletion affecting the reading frame, even though the region is present in SfNicB and other Group II alphabaculoviruses. In fact, in that location was annotated other ORF (SfNicB ORF130) with similarity with AcMNPV ORF29 and SeMNPV ORF128. It is important to note that homologs of SfNicB ORF130 are also present in the other genotypes of SfMNPVs, including SfColA, and were annotated as ORF130 in Sf3AP2 and ORF128 in Sf19. For these reasons, in the genome of SfColA both putative coding regions were included as ORF131 and ORF131a (Fig. 1e).

Regarding non-encoding *loci*, baculoviruses *homologous regions* (*hr*) are sequence repeats which are dispersed throughout their genomes. All previously described SfMNPVs have 8 h interspersed in different locations; they are characterized by tandem repeats consisting of a 44 bp nucleotide stretch which include an imperfect 34 bp palindromic core. These sequences are variable; however, the local secondary structure motifs are very similar, constituting hairpin loops (see Additional file 2: Figure S1). The *hr*-1 has 7 repeats; *hr*-2, *hr*-3 and *hr*-6 have only 1 repeat; *hr*-4 and *hr*-7 have 6 repeats and *hr*-5 and *hr*-8 have 4 repeats. It should be noted that SfColA lacked *hr*-3 since this sequence was located in the region where gene replacement occurred (Fig. 1f). Sf19 lacked *hr*-5c and *hr*-5d, and SfNicB lacked *hr*-8a and *hr*-8b. Two unique ORFs (039a and 110a) were annotated in SfNicB but the corresponding sequences were also present in Sf19, Sf3AP2 and SfColA showing few single nucleotide polymorphisms. The *locus* containing SfNicB ORF039a was located close to *hr-*4 while the SfNicB ORF110a was close to *hr-*7 and both postulated encoding sequences were probably not real genes. All repeats from SfMNPV *hr*s can be summarized in a consensus sequence using the IUPAC ambiguity code: 5′ YN**A**WS**TT**DR**CTTT**YVDYN**A**HRHDYB**T**BRNBD**AAA**KYM**AA**SW**T**BR 3′. Conserved nucleotides (bold) would be A or T and probably essential for their role as replication origins and/or as transcription enhancers.

**Fig. 2:**
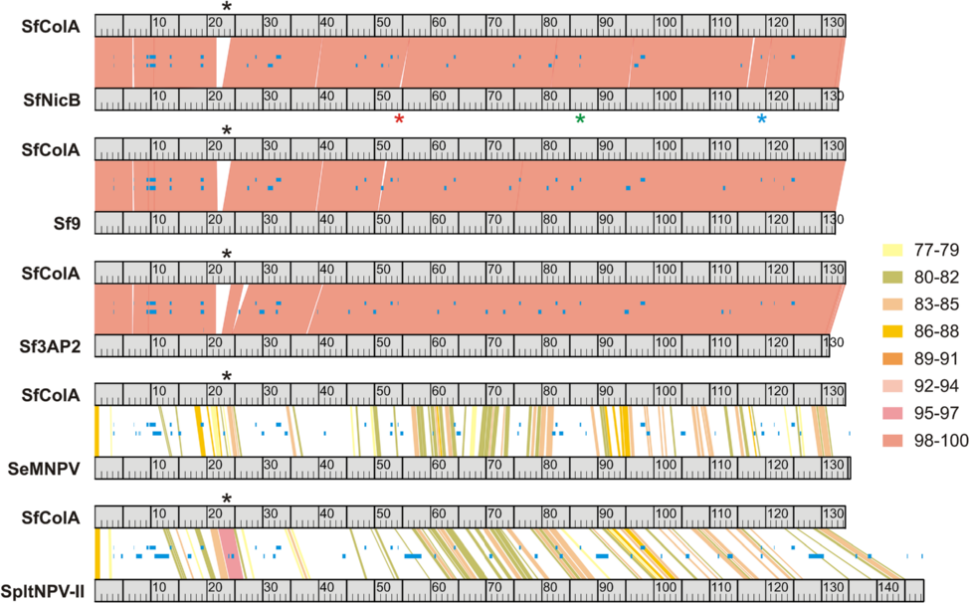
Nucleotide genome synteny. Individual genome comparisons between SfMNPV ColA (SfColA) and the other genotypes of this baculovirus species [SfMNPV NicB (SfNicB), SfMNPV 19 (Sf19) and SfMNPV 3AP2 (Sf3AP2)], SeMNPV and SpltNPV-II are shown. Genome sizes are represented as rules and a colored key is used to show similarity percentages. The *locus* where SfColA ORF023/024 are located is depicted as a black asterisk. The other asterisks indicate the locations where SfNicB has insertions [309 bp (red), 73 bp (green) and 60 bp (blue)]. Blue blocks in the middle of each graph indicate A + T rich regions

Previous results were confirmed by a genome colinearity study showing high nucleotide sequence conservation and genome organization among SfMNPV genotypes (Fig. 2). The exceptions included the *locus* where SfColA lost a ~1470 bp fragment and acquired another one of ~2970 bp (ORFs 023 and 024), being similar to SpltNPV-II regions, and 3 small insertions present in only SfNicB. The first was 309 bp, located downstream to the *odv-e66* gene and producing SfNicB ORF057a. The second insertion was 73 bp, located in the intergenic region of SfNicB ORFs 085 and 086, and the third one was 60 bp positioned 437 bp upstream SfNicB ORF131 (*p26* gene) (Fig. 1e).

**Fig. 3:**
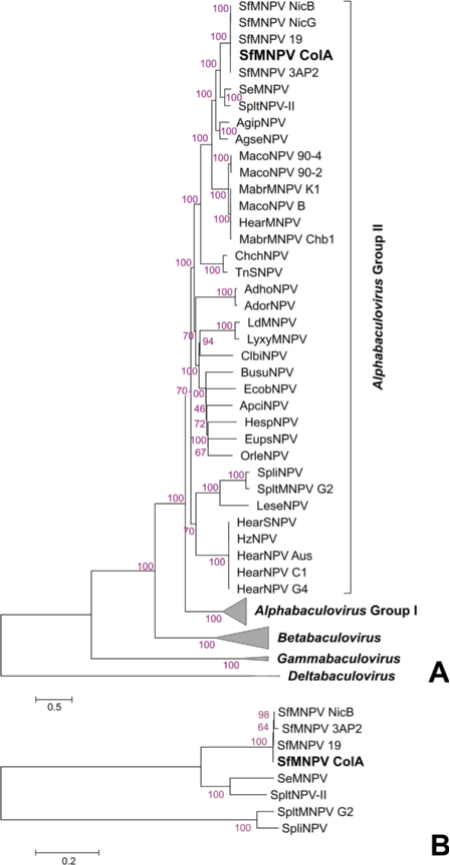
Phylogenetic inference for SfMNPV ColA. **a** Cladogram based on a concatemer built with the 37 core proteins obtained from 74 baculoviral genomes and SfMNPV ColA. The phylogenetic tree was inferred using the MEGA 5 program. The four *Baculoviridae* genera are indicated and *Alpha*- (Group I), *Beta*- and *Gammabaculovirus* clades were collapsed to preserve space. **b** Cladogram based on a concatemer built with 100 homologous proteins obtained from 8 baculoviral genomes including SfMNPV ColA. The phylogenetic tree was inferred using the MEGA 5 program

Phylogenetic analysis was based on 37 concatenated core proteins derived from 75 baculovirus genomes (Fig. 3a); as expected, the cladogram reproduced the grouping in 4 genera recognized in the current classification of the virus family [2]. SfColA and the other SfMNPVs formed a clade which was included in Group II *Alphabaculovirus,* the closest species being SeMNPV and SpltNPV-II. Special attention should be paid to other baculovirus isolates recovered from the same insect species, such as *Spodoptera litura*, *Mamestra configurata*, *Mamestra brassicae*, *Helicoverpa armigera*, *Helicoverpa zea*, *Agrotis ipsilon* and *Agrotis segetum* NPVs. By contrast with SfMNPV, some members of the previously mentioned set of viruses grouped in different clades, thereby reflecting their greater diversity. Another inference was made regarding phylogeny, but only using the most closest related viruses based on 100 concatenated orthologous proteins (Fig. 3b). This study has revalidated the consistency of SfMNPV relationships with SeMNPV and SpltNPV-II (the closest baculovirus species) and has also highlighted the difficulty of finding groupings among the different genotypes of baculoviruses infecting *Spodoptera* spp. since non-orthologous proteins were not included in these studies.

### Interspecies horizontal gene transfer

**Fig. 4:**
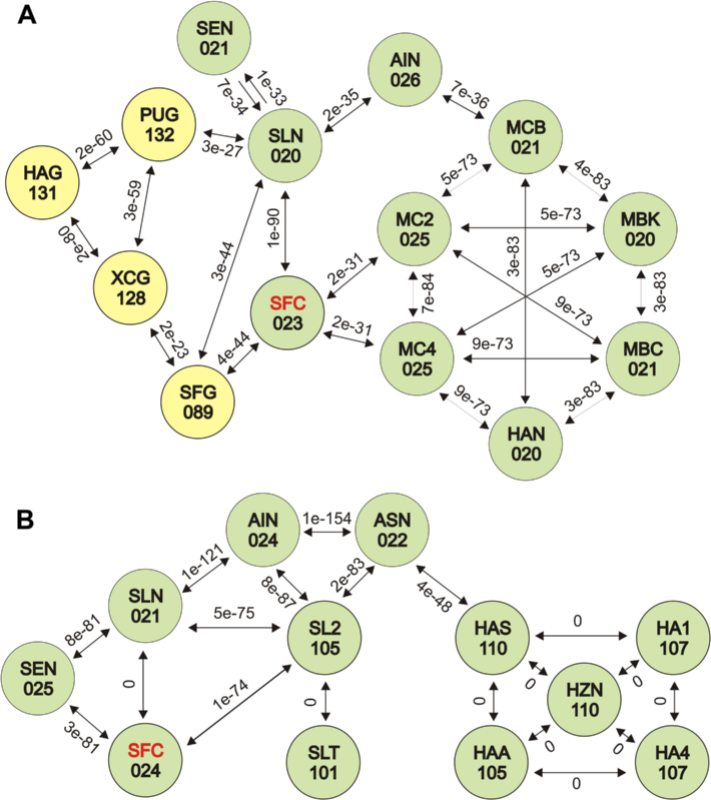
Protein relationships for SfMNPV ColA ORFs 023 and 024. The relationships among two SfMNPV ColA ORFs and their orthologs contained in other viruses were calculated by BlastP. Related baculovirus species are shown (three letter abbreviations for species and ORF number) in filled circles (yellow for betabaculoviruses and green for Group II alphabaculoviruses). The BlastP e-value between pairs of species is indicated above each arrow. **a** Protein relationships for SfMNPV ColA ORF 023. **b** Protein relationships for SfMNPV ColA ORF 024. AIN: AgipNPV; ASN: AgseNPV; HAA: HearNPV Aus; HA1: HearNPV C1; HA4: HearNPV G4; HAG: HearGV; HAN: HearMNPV; HAS: HearSNPV; HZN: HzSNPV; MC2: MacoNPV 90–2; MC4: MacoNPV A90-4; MBC: MabrNPV CHb1; MBK: MabrNPV K1; MCB: MacoNPV B; PUG: PsunGV; SEN: SeMNPV; SFC: SfMNPV ColA (in red letters); SFG: SpfrGV VG008; SL2: SpltMNPV G2; SLN: SpltNPV-II; SLT: SpliNPV AN1956; XCG: XecnGV

The most important difference among SfMNPVs was the sequence acquisition which occurred in SfColA genome; this involved acquiring two genes from other baculovirus species and the loss of one gene present in all remaining molecularly characterized SfMNPV. A detailed study aimed at determining orthology with other baculoviruses showed that SfColA ORF023 and ORF024 were closely related to annotated SpltNPV-II ORFs (Fig. 4). These sequences from both genomes had higher than 95 % identity and similarity, similar to the value when comparing homologous proteins between pairs of SfMNPV genotypes (Additional file 1: Table S1). The study revealed that the ORF023 had putative orthologs in Group II alpha- and betabaculoviruses while ORF024 had homologs only in Group II alphabaculoviruses.

**Fig. 5:**
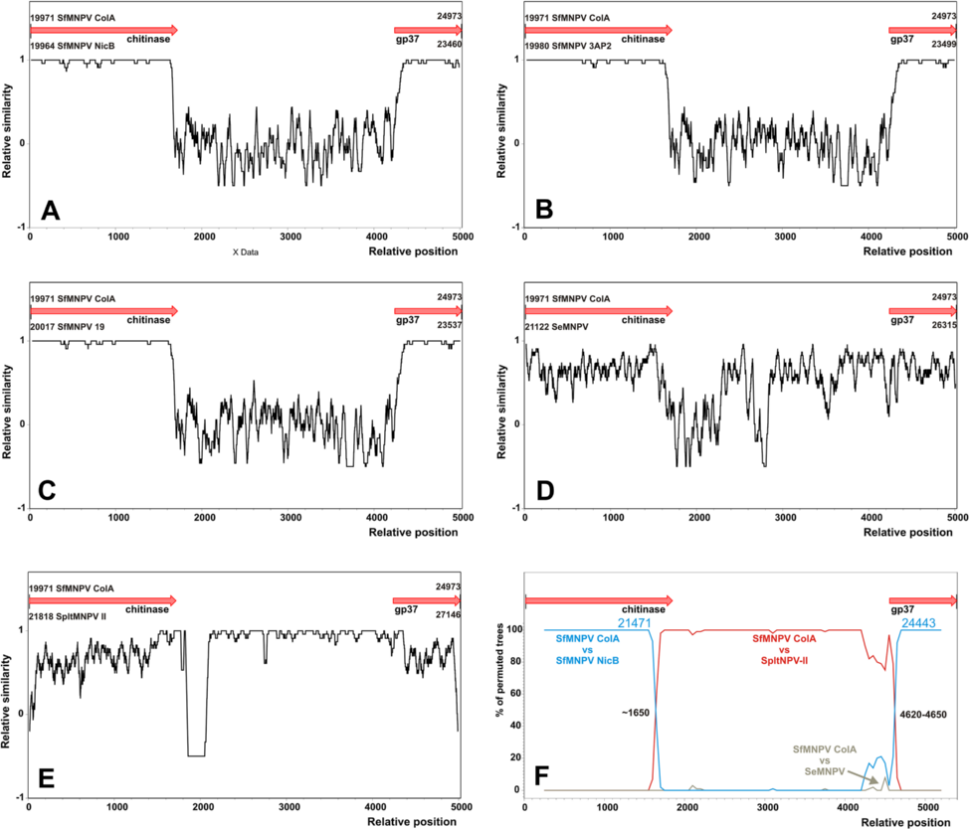
SfMNPV ColA ORFs 023 and 024 origin by horizontal transfer. The recombination process between SfMNPV ColA and SpltNPV-II ancestors was studied by similarity plots and bootscanning analysis. Genome regions analyzed were those containing SfMNPV ColA ORFs 023 and 024, *chitinase* and *gp37* genes. In all cases, colored arrows represent SfMNPV ColA ORFs. Genome positions are indicated at the beginning and the end of the regions analyzed (bp scale). Similarity plots are indicated in black, and different colors (explained in the graphs) are used. **a** Similarity plot between SfMNPV ColA and SfMNPV NicB. **b** Similarity plot between SfMNPV ColA and SfMNPV 3AP2. **c** Similarity plot between SfMNPV ColA and SfMNPV 19. **d** Similarity plot between SfMNPV ColA and SeMNPV. **e** Similarity plot between SfMNPV ColA and SpltNPV-II. **f** Bootscanning using SfMNPV ColA, SfMNPV NicB, SeMNPV and SpltNPV-II

Different approaches were used to explore the recombination hypothesis regarding recent SfMNPV and SpltNPV-II ancestors. The first one consisted in a relative similarity analysis between the genome region involved in the structural mutation from SfColA and the other SfMNPVs, SeMNPV and SpltNPV-II. Poor similarity was revealed regarding the sequences in all the other SfMNPVs, although the *chitinase* (upstream region) and *gp37* (downstream region) genes were almost identical (Fig. 5a–c); by contrast, similarity increased when compared with SeMNPV (Fig. 5d) and reached the maximum value with SpltNPV-II (Fig. 5e). It is worth noting that the only SfColA region regarding the other SfMNPVs was very closely related to SpltNPV-II but that the upstream and downstream sequences had lower similarity values. Another approach based on bootscaning analysis validated previous results showing that a recent ancestor of SpltNPV-II was the most probable DNA donor involved in recombination (Fig. 5f).

**Fig. 6:**
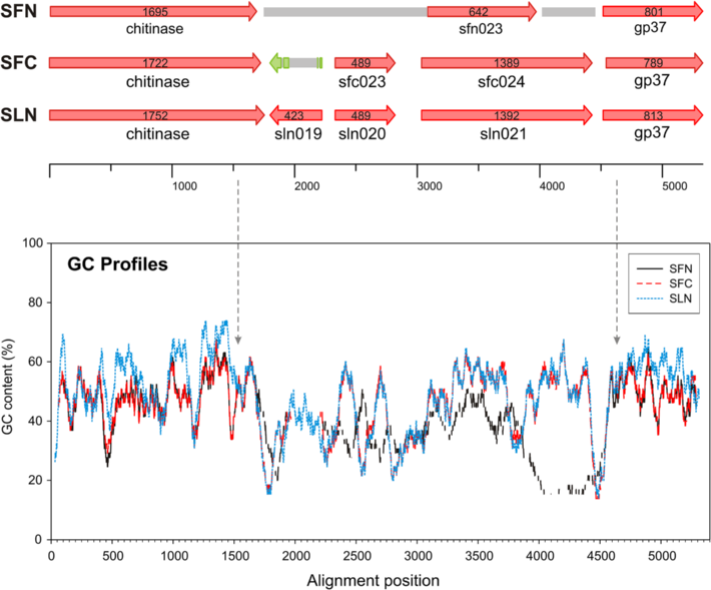
G + C profile into the *locus* containing SfMNPV ColA ORFs 023 and 024. The G/C-contents (%) of SfMNPV ColA, SfMNPV NicB and SpltNPV-II were analyzed in the *locus* of each genome where the recombination process occurred. Profiles are shown with different colors referenced in the graph. A representation of coding regions (indicated as arrows) is featured above the histogram. Grey boxes do not represent sequences and are used to facilitate understanding the graphical positions of *chitinase* and *gp37* genes among analyzed genomes

A G/C-content study was performed to demonstrate gene transfer by transposition in SeMNPV [25]. Such approach not based on sequence alignments providing similar results to those aforementioned (Fig. 6). The recombinant region’s G/C profile was more similar to SpltNPV-II than to the other SfMNPVs (43.3 % G/C-content in SfColA and 43.1 % in SpltNPV-II compared to 35.7 % in SfNicB/Sf19/Sf3AP2). By contrast, upstream and downstream regions (*chitinase* and *gp37* genes) had a completely different pattern, having similar values to the G/C-content average (40.3 % in SfMNPVs compared to 45 % in SpltNPV-II). Kimura 2-parameter (K-2-P) distances were estimated to further support the idea of recent recombination (Tables 2 and 3). This approach revealed a very close relationships between SFColA ORF023 and ORF024 regarding SpltNPV-II ORF020 and ORF021, respectively, expressing distances larger than 0.015 but smaller than 0.050. This range of values is currently assumed as an interval in what complementary information is needed to determine whether two sequences are different or genotypes of the same species [1]. The present study thus revealed that the sequences flanked by *chitinase* and *gp37* genes in SfColA and SpltNPV-II genomes would belong to baculovirus isolates from the same species; by contrast, the other genes from both genomes were revealed to be sequences of two different baculovirus species.

**Table 2:**
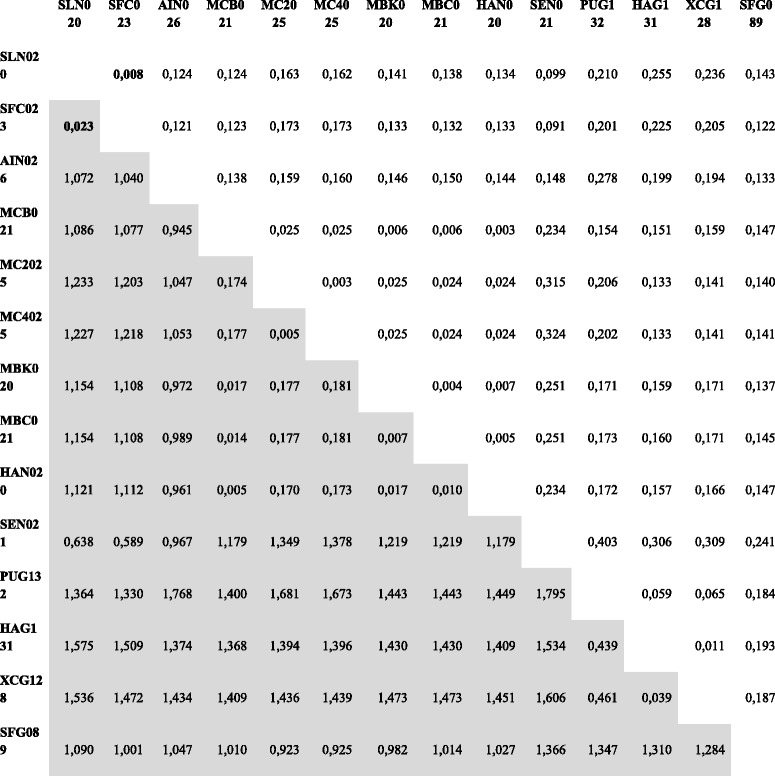
Kimura 2-parameter distances between ORFs 020/021 of SpltNPV-II and their corresponding orthologs

The K-2-P values are shaded in gray (lower triangles). The other values are the corresponding standard errors (upper triangles)

*SLN* SpltNPV-II, *SFC* SfColA, *AIN* AgipNPV, *MCB* MacoNPVB, *MC2* MacoNPV 90-2, *MC4* MacoNPV 90-4, *MBK* MabrMNPV K1, *MBC* MabrMNPV Chb1, *HAN* HearMNPV, *SEN* SeMNPV, *PUG* PsunGV, *HAG* HearGV, *XCG* XecnGV, *SFG* SpfrGV, *HAS* HearsNPV, *HAA* HearNPV Aus, *HA1* HearNPV C1, *HA4* HearNPV G4, *HZN* HzSNPV, *ASN* AgseNPV, *SL2* SpltMNPV G2, *SLT* SpliNPV AN1956

**Table 3:**
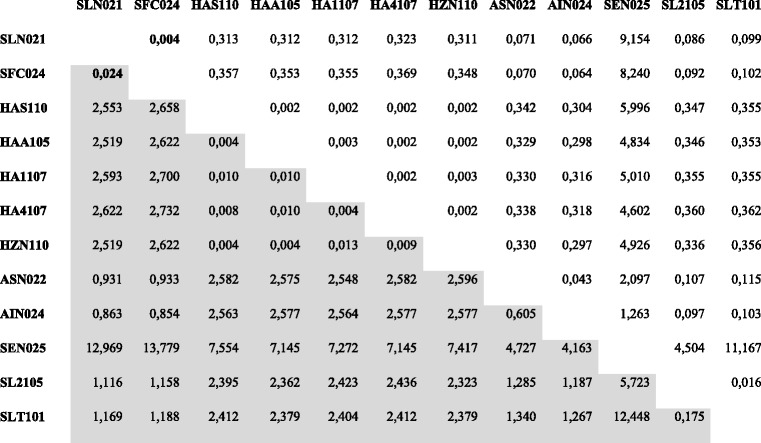
Kimura 2-parameter distances between ORFs 020/021 of SpltNPV-II and their corresponding orthologs

The K-2-P values are shaded in gray (lower triangles). The other values are the corresponding standard errors (upper triangles)

*SLN* SpltNPV-II, *SFC* SfColA, *AIN* AgipNPV, *MCB* MacoNPVB, *MC2* MacoNPV 90-2, *MC4* MacoNPV 90-4, *MBK* MabrMNPV K1, *MBC* MabrMNPV Chb1, *HAN* HearMNPV, *SEN* SeMNPV, *PUG* PsunGV, *HAG* HearGV, *XCG* XecnGV, *SFG* SpfrGV, *HAS* HearsNPV, *HAA* HearNPV Aus, *HA1* HearNPV C1, *HA4* HearNPV G4, *HZN* HzSNPV, *ASN* AgseNPV, *SL2* SpltMNPV G2, *SLT* SpliNPV AN1956

All the aforementioned analysis suggested that recombination occurred between recent SfMNPV and SpltNPV-II ancestors, involving the end of the *chitinase* gene and the start of the *gp37* gene, causing the replacement of ~1470 bp (including *hr*-3 and SfNicB/Sf3AP2 ORF023 or Sf19 ORF022) for ~2970 bp carrying 2 complete genes having great similarity to SpltNPV-II 020 and 021 ORFs and a truncated gene similar to SpltNPV-II ORF019. Breakpoints seemed to be inside the reading frames for the *chitinase* gene (around the position 21,471 in SfColA) and the *gp37* gene (around the position 24,443 in SfColA) restoring the continuity of both frames. Regarding the SpltNPV-II ORF019 homolog in SfColA, a sequence analysis revealed 8 different deletions (11 bp, 240 bp, 11 bp, 3 bp, 1 bp, 3 bp, 1 bp and 11 bp, ordered from ATG to stop codon) thereby resulting in a frame shift.

### SfColA ORF023 and ORF024

**Fig. 7:**
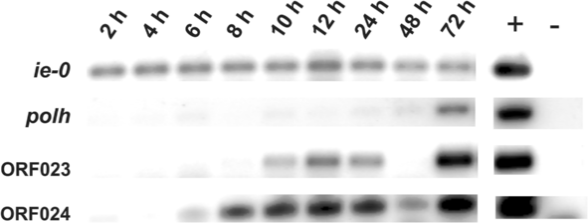
Transcription kinetics of SfMNPV ColA ORFs 023 and 024. *Spodoptera frugiperda* larvae were exposed to SfMNPV ColA and whole RNAs were isolated from sacrificed animals at different intervals post-infection. Then, cDNA with polyT primer was generated for each sample and PCR assays were done using specific primers which amplify fragments of different ORFs from SfMNPV ColA genome [*polyhedrin* (*polh*), *immediate-early 0* (*ie-0*), ORF023 and ORF024]. Figure shows a photo cut-out showing the amplification bands resolved by agarose gel electrophoresis. The SfMNPV ColA genome was used as positive control and water was used as negative control

Whole RNAs isolated at different times from *S. frugiperda* larvae orally infected with SfColA were examined by reverse transcription PCR to determine whether SfColA ORF023 and ORF024 were active transcriptional units (Fig. 7). The very late SfColA ORF001 (*polyhedrin*) and the inmediate-early SfColA ORF143 (*ie-0*) were included for reference. Single RT-PCR products were obtained having the expected sizes (214 bp -ORF023-, 166 bp -ORF024-, 255 bp -ORF001- and 163 bp -ORF143-). This experimental approach showed that transcripts from ORF023 appeared at 10 *hpi* while ORF024 started at 6 *hpi*, a result in agreement with the presence of early INR promoter motifs (Additional file 1: Table S1).

**Fig. 8:**
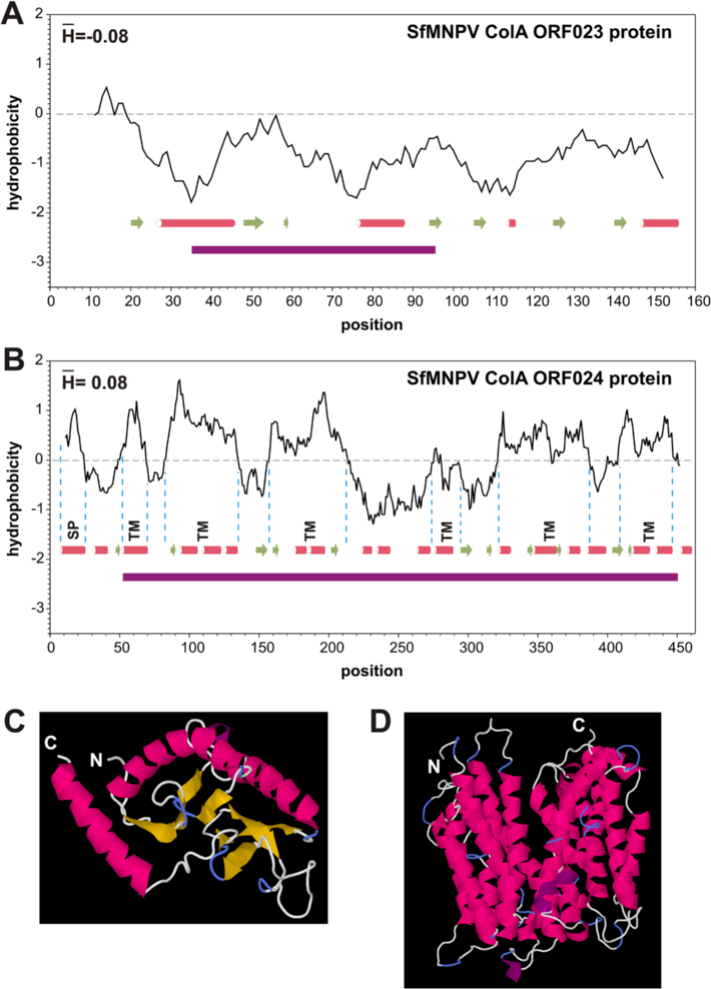
Characterization of theoretical proteins derived from SfMNPV ColA ORFs 023 and 024. The theoretical proteins encoded by SfMNPV ColA ORFs 023 (panels **a** and **c**) and 024 (panels **b** and **d**) were analyzed and the 3D structures were predicted. Hydrophobicity profiles and predicted secondary structures are shown. Alpha helices are represented as red cylinders and beta sheets as green arrows. The putative signal peptide (SP) and transmembrane domains (TM) are also shown

Predictive studies were then performed for SfColA ORF023 and ORF024 proteins. The SfColA ORF023 theoretical polypeptide thus consisted of 162 residues, 19 being negatively charged (Asp + Glu) and 29 positively charged amino acids (Arg + Lys + His). Based on sequence, the molecular weight is 19,087.1 Da and the theoretical isoelectric point is 9.39. The hydrophobicity profile suggested that this polypeptide was a soluble protein having average hydrophobicity of −0.08. The secondary structure predicted by the LOMETS and I-TASSER servers gave 85.8 % coincidence, revealing the presence of 3 α-helices (28.4 % of residues), 5 β-sheets (17.3 % of residues), and the remaining amino acids constituting loops or turns (Fig. 8a). The QUARK server predicted that ORF023 would be a globular protein having a tertiary structure according to previous results (Fig. 8b). HHpred identified a region (from amino acid 35 to 95) as being a phosphatase domain similar to *Schizosaccharomyces pombe* Polynucleotide kinase 3 phosphatase (PNK1; [59]) which plays a role in repairing single breaks in DNA induced by several DNA-damaging agents. INTERPROSCAN identified 2 protein kinase C phosphorylation sites (from amino acids 66 to 68 and from amino acid 75 to 77) and 1 tyrosine-kinase phosphorylation site (from amino acid 97 to 104). These post-translational modifications could be part of activation/inactivation processes, but require experimental confirmation.

The SfColA ORF024 theoretical protein consisted of 462 residues, 33 being negatively charged (Asp + Glu) and 41 positively charged amino acids (Arg + Lys + His). Based on sequence, the molecular weight was 52,210.0 Da and the theoretical isoelectric point was 7.60. The hydrophobicity profile suggested that this polypeptide would be a membrane protein having +0.08 average hydrophobicity and having at least 6 transmembrane regions containing 12 α-helices and a signal peptide detected by SignalP (Fig. 8c). The secondary structure predicted by LOMETS and I-TASSER servers gave 85.8 % coincidence, revealing the presence of 19 α-helices (47.8 % of residues), 11 β-sheets (12.5 % of residues), and the remaining amino acids constituting loops or turns. Coincidentally, the LOMETS server predicted a tertiary structure and a secondary motif distribution consistent with a transmembrane motif (Fig. 8d). HHpred identified one region (from amino acid 51 to 451) as a member of the Major Facilitator Superfamily (MFS); these proteins are permeases which act as secondary carriers in cell transport [60]. INTERPROSCAN found several putative post-translational modifications, including 2 N-glycosylation sites (from amino acid 36 to 39 and from amino acid 317 to 320), 1 cAMP- and cGMP-dependent protein kinase phosphorylation site (from amino acid 3 to 6), 2 Protein kinase C phosphorylation sites (from amino acid 452 to 454 and from amino acid 455 to 455) and 1 Casein kinase II phosphorylation site (from amino acid 50 to 53). These post-translational modifications could form part of protein function but experimental confirmation is required. It should be mentioned that the homologous protein encoded by the *Helicoverpa armigera* nucleopolyhedrovirus (G4 strain) was not detected as a structural protein, suggesting that its role occurs within the infected cells [61, 62].

It is importante to note that all the data sets supporting the results of this article are included within the article and its additional files.

## Conclusions

Baculoviruses and other viruses having large dsDNA genomes mainly evolve due to the accumulation of structural mutations (insertions, deletions, replacements, inversions, translocations) affecting gene content, where recombination or transposition appear to be the most relevant examples of mechanisms occurring in nature affecting DNA integrity. Analysis of complete baculovirus genomes has revealed a “core genome” represented by 37 genes encoding essential factors accumulating sequence variability since the last virus ancestor [58]. Such pathogens carry sequences acquired from other entities defining a “plastic genome” which contains sequences included in all members of each genus and other regions present in only some species or variants. Core genes usually produce key factors needed to complete a virus cycle, by contrast many encoding sequences in the plastic genome produce auxiliary proteins collaborating in virus processes even though not being essential for producing infectious progeny increasing fitness for them to perpetuate in nature. New technologies available for acquiring whole genome information have facilitated associating phenotype characteristics with gene content. The SfMNPV ColA genome (from a particular Colombian isolate having better biological properties than others) [21] was thus sequenced having high coverage and compared to other genotypes isolated from other geographical regions.

The most relevant differences occurred in a *locus* where SfColA underwent recent sequence replacement, losing 1 gene and gaining 2 new encoding sequences. The genome location where recombination occurred has been described as hypervariable since SfMNPV variants have different deletions [17, 18, 21]. These regions include auxiliary genes such as *ecdysteroid UDP–glucosyltransferase* (*egt*), *protein-tyrosine-phosphatase* (*ptp*), *chitinase* and *cathepsin* whose products have activity affecting insect host physiology, development, behavior and integrity [20]. Interestingly this location also contains *hr-*3, a kind of sequences recognized as being a recombination facilitators [27, 28]. By contrast, most of the other *hr*s were closer to core genes, thereby decreasing the fitness of natural recombinant viruses due to possible loss of essential functions. It is worth stressing that *hr-*1 was close to *odv-56* (*pif5*) and *f* genes, *hr-*2 was near *lef-1*, *hr-*4 next to *alk-exo*, *hr-*6 was close to *lef-9* and *hr-*7 was near *lef-8* and *u-box/ring*. The *locus* containing *hr-*3 thus seemed to be a hot genome region prone to undergoing structural mutations.

Recombination is an important evolutionary mechanism which might be used as a viral strategy to gain advantage for maintaining adaptability to changing environments [27]. Recombination could facilitate resistance to host range expansion [63–65]. These kinds of interactions between genomes occur if two types of DNA coexist in the same cell, have sequence similarities and they are replicating. Artificial coinfections with AcMNPV and BmNPV in larvae and in cell culture have shown that homologous recombination can occur between viruses belonging to two different species [66]. It has been reported that some SpfrGV genes were acquired by horizontal gene transfer from other baculovirus species including SpltNPV-II [37], such genome having been identified as DNA donor for SfColA ORFs 023 and 024. SpfrGV contains an orthologous gene for SfNicB ORF023, Sf19 ORF022 and Sf3AP2 ORF023 (SpfrGV ORF099), the encoding sequence lost in SfColA. This would suggest that recombination occurred when these viruses co-infected *Spodoptera frugiperda* larvae.

*Spodoptera litura* and *Spodoptera frugiperda* are polyphagous insect pests living on crops such as rice, corn, cotton and tobacco; they have been reported in subtropical locations in both the Old and New world, although cross migration of both insects has been reported [67]. *S. litura* have been recorded in 80 species of host plant [68] while *S. frugiperda* has been described in 186 such plants [69], many of them shared between both lepidoptera. Natural coinfection involving circulating variants of SpltNPV and SfMNPV could thus occur in the same host. Both species have similar sequences and genome organization, and it has been reported that SpltNPV can infect *Spodoptera frugiperda*-derived cells, such as Sf9 and Sf21 [65, 68]. The above and bioinformatics evidence provided here support the hypothesis that homologous recombination is used by baculoviruses in nature to acquire variability. The SfColA genome would thus seem to provide natural proof for affirming that horizontal gene transfer is exploited by organisms and viruses to increase their fitness and thus acquire a reproductive success ensuring their permanence in nature.

## Additional files

Additional file 1: Table S1. Potentially expressed ORFs in the genome of SfMNPV ColA. In bold letter are indicated the core genes. (XLSX 32 kb) Additional file 2: Figure S1. Sequence comparisons and structural analysis of homologous regions. The palindromes contained into the homologous regions (*hr*) from SfMNPV ColA were analyzed and the consensus sequences represented by Sequence logos. Besides, predictions of the ssDNA secondary structures are shown. 1-A. *hr*-1 (28 palindromes). 1-B. *hr*-2 (4 palindromes). 1-C. *hr*-3 (3 palindromes). 1-D. *hr*-4 (24 palindromes). 1-E. *hr*-5 (14 palindromes). 1-F. *hr*-6 (4 palindromes). 1-G. *hr*-7 (24 palindromes). 1-H. *hr*-8 (14 palindromes). 1-I. All palindromes (115 repeats). 2. Largest A + T-rich region in the SfMNPV ColA genome. 2-A. Physical map of *hr*-1 and location of the largest A + T-rich region. 2-B. Nucleotide sequence detail. 2-C. Local ssDNA secondary structure. (TIF 3246 kb)
