# Supplementary figures and images for: Evidence of recent interspecies horizontal gene transfer regarding nucleopolyhedrovirus infection of Spodoptera frugiperda
Source: BMC Genomics. 2015 Nov 25;16:1008. doi: 10.1186/s12864-015-2218-5 (PMC4861128; doi:10.1186/s12864-015-2218-5)

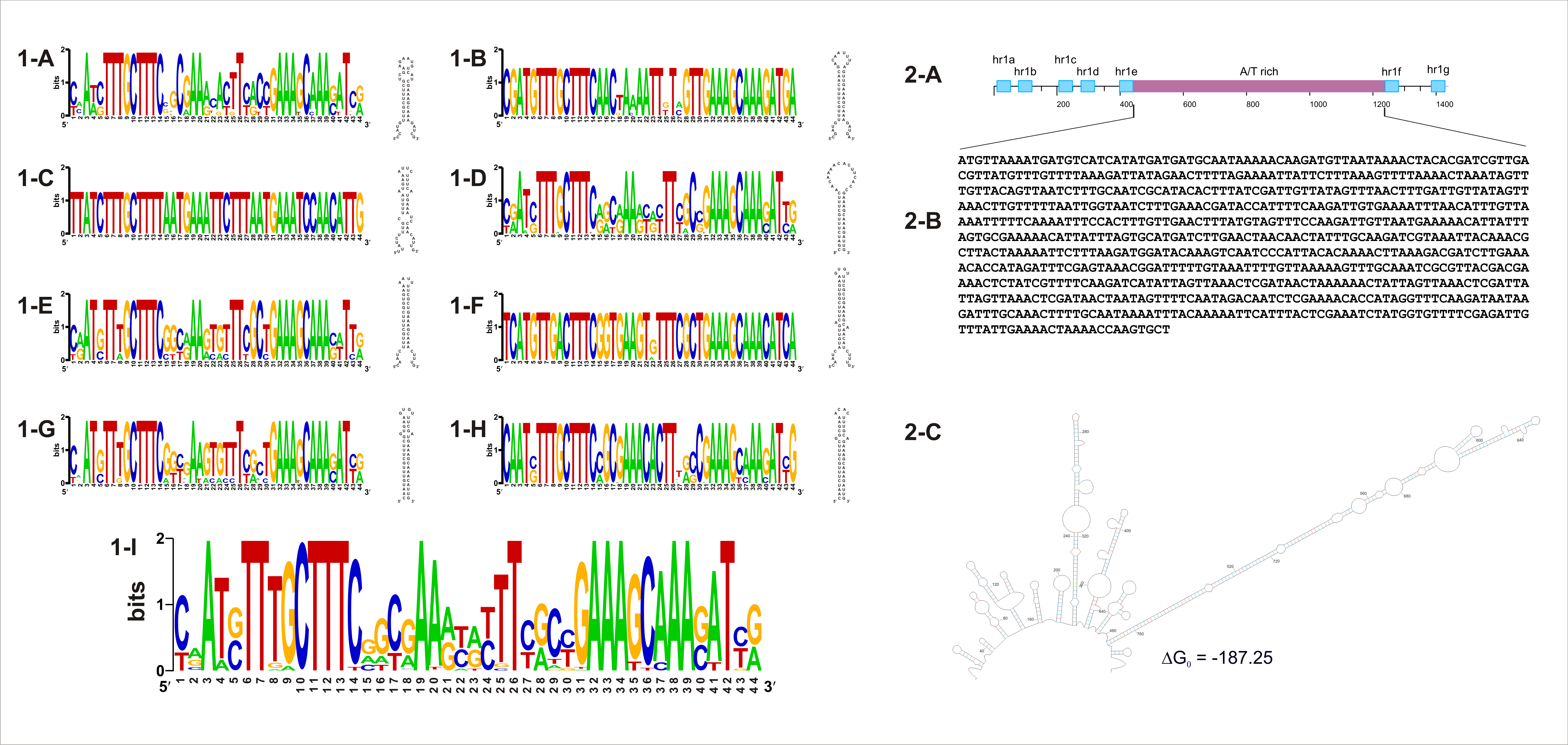

Supplement: Additional file 2: Figure S1. — Sequence comparisons and structural analysis of homologous regions. The palindromes contained into the homologous regions (hr) from SfMNPV ColA were analyzed and the consensus sequences represented by Sequence logos. Besides, predictions of the ssDNA secondary structures are shown. 1-A. hr-1 (28 palindromes). 1-B. hr-2 (4 palindromes). 1-C. hr-3 (3 palindromes). 1-D. hr-4 (24 palindromes). 1-E. hr-5 (14 palindromes). 1-F. hr-6 (4 palindromes). 1-G. hr-7 (24 palindromes). 1-H. hr-8 (14 palindromes). 1-I. All palindromes (115 repeats). 2. Largest A + T-rich region in the SfMNPV ColA genome. 2-A. Physical map of hr-1 and location of the largest A + T-rich region. 2-B. Nucleotide sequence detail. 2-C. Local ssDNA secondary structure. (TIF 3246 kb) [file 12864_2015_2218_MOESM2_ESM.tif]
